# Exploring implementation practices in results-based financing: the case of the verification in Benin

**DOI:** 10.1186/s12913-017-2148-9

**Published:** 2017-03-14

**Authors:** Matthieu Antony, Maria Paola Bertone, Olivier Barthes

**Affiliations:** AEDES Consulting, Rue Joseph II 34, Brussels, 1000 Belgium

**Keywords:** Performance-based financing, Results-based financing, Verification, Implementation, Benin

## Abstract

**Background:**

Results-based financing (RBF) has been introduced in many countries across Africa and a growing literature is building around the assessment of their impact. These studies are usually quantitative and often silent on the paths and processes through which results are achieved and on the wider health system effects of RBF. To address this gap, our study aims at exploring the implementation of an RBF pilot in Benin, focusing on the verification of results.

**Methods:**

The study is based on action research carried out by authors involved in the pilot as part of the agency supporting the RBF implementation in Benin. While our participant observation and operational collaboration with project’s stakeholders informed the study, the analysis is mostly based on quantitative and qualitative secondary data, collected throughout the project’s implementation and documentation processes. Data include project documents, reports and budgets, RBF data on service outputs and on the outcome of the verification, daily activity timesheets of the technical assistants in the districts, as well as focus groups with Community-based Organizations and informal interviews with technical assistants and district medical officers.

**Results:**

Our analysis focuses on the actual practices of quantitative, qualitative and community verification. Results show that the verification processes are complex, costly and time-consuming, and in practice they end up differing from what designed originally. We explore the consequences of this on the operation of the scheme, on its potential to generate the envisaged change. We find, for example, that the time taken up by verification procedures limits the time available for data analysis and feedback to facility staff, thus limiting the potential to improve service delivery. Verification challenges also result in delays in bonus payment, which delink effort and reward. Additionally, the limited integration of the verification activities of district teams with their routine tasks causes a further verticalization of the health system.

**Conclusions:**

Our results highlight the potential disconnect between the theory of change behind RBF and the actual scheme’s implementation. The implications are relevant at methodological level, stressing the importance of analyzing implementation processes to fully understand results, as well as at operational level, pointing to the need to carefully adapt the design of RBF schemes (including verification and other key functions) to the context and to allow room to iteratively modify it during implementation. They also question whether the rationale for thorough and costly verification is justified, or rather adaptations are possible.

## Background

Results-based financing (RBF), also called Performance-Based Financing or Pay for Performance [1], is increasingly being piloted and implemented at national level in numerous countries across sub-Saharan Africa, and it is the subject of much interest and debate in terms of the assumptions on which it is based as well as its potential impact to improve health outcomes [2–4]. Although evidence on the effects of RBF was considered insufficient until recently [5], a rigorous program of impact evaluations mainly through randomized control trials (RCTs) has been put in place for many schemes during the pilot stage. This research has led to the production of an expanding body of literature [6–10]. While RCTs and other quantitative methods are useful to better understand the effects of RBF on health worker motivation and health outputs or outcomes, they are often silent on the paths and processes through which these results are achieved (beyond the hypotheses that they set off to test), and on the wider health system effects of the schemes [11]. As shown by Ssengooba et al. [12], it is essential to couple those studies with detailed qualitative or mixed-methods analysis to open the ‘black box’ between intervention and results, and assess how the design and the implementation of the scheme affect its potential impact on health outcomes and may have systemic effects, thus defining the success of the intervention. Not only implementation processes are known to influence the outcomes of policies and interventions [13], but the contextual features of the implementation are key to understand such processes and their outcomes, and deserve to be analyzed in depth [14]. For this analysis, it is important to focus on those components that critically contribute to the functioning of a scheme and are key in the theory of change of RBF [15].

Despite the variety of labels used, the term ‘results-based financing’ generally refers to schemes which entail a transfer in resources when some form of performance criteria is met [11]. In this paper, we make reference to RBF schemes that focus on the supply-side, target bonuses both to facilities and individual providers and where payments are linked to the quantity of outputs produced, modified by quality indicators [2, 11, 16, 17]. In such schemes, in practice, facilities sign a contract in which the terms of the scheme are defined, and rules, payments and sanctions established. Based on that, once facilities have provided health services, they send an invoice to a purchasing agency (either an external implementation agency or a governmental body) in which they request payment of the agreed (fee-for-service) bonus for the sub-set of services provided which are included in the RBF scheme. Such invoice is then verified, and the quality of the healthcare environment also assessed in order to calculate and make a payment to the facility. The use of the RBF bonus is autonomously defined by the facility staff, to both improve the working environment and incentivize individual staff. Therefore, as pointed out by Witter et al [11], RBF is based on the assumption that individuals and organizations are motivated to perform better by financial incentives, and that better results can be promoted by linking payments to desired outputs and encouraging decision autonomy and entrepreneurial behavior at facility level. This is done by revising institutional arrangements, clarifying roles and tasks of each actor and establishing a set of explicit contractual relations, which define rewards and sanctions, as well as verification and enforcement mechanisms.

Obviously, the verification of results plays a key role in such schemes and has indeed been termed a ‘cornerstone’ of RBF programs [16]. Verification ensures that services, for which a payment request (invoice) is made, have been actually provided and that they are of good quality [16]. The rational for carrying out a detailed verification of both quality and quantity of services provided lies, first of all, in the practical necessity of calculating the reward (i.e. financial bonus) accrued by facilities and pay them a bonus in a transparent manner (which enhances their trust), as well as promptly and regularly based on their effort and performance. It also entails the opportunity for detecting frauds and for signaling to providers a real threat of sanction in case of irregularities, such as gaming on quantity of services, lowering quality of services and reducing patients’ satisfaction. Ideally, verification of results creates positive spill-over effects also at system-level. If the verification procedures include a patient satisfaction survey (as it is the case in Benin), they could also be seen to play an important role to channel the “voice” of the communities, which may in theory allow for increased provider accountability [16, 18]. Finally, strong and reliable verification mechanisms can improve the quality of the routine information system by both contributing to a change in providers’ views on data reporting [2], and by providing a verified comparison to assess it. Importantly, the availability of information on performance allows for the possibility of data analysis on a monthly or quarterly basis. It has been noted that facility staff and managers appreciate and are responsive to having detailed feedback on their performance [3]. This is even more useful if accompanied by supervision (by district health authorities) and coaching (by implementing agency) to identify the issues limiting the facility’s performance as well as of strategies to address them. The latter is another key element of RBF, because with increased autonomy, providers need more data and (at least initially) external support for decision-making. Additionally, the involvement of district health authorities in the verification procedures, accompanied by their analysis of district-level data can reinforce governance and stewardship of the system and improve the management of drugs, equipment and human resources [19]. However, it is important to stress that, the key role potentially played by the verification of results in RBF should not overshadow its costs. Indeed, as argued for RBF overall [20], the verification processes should be financially viable, so that their benefits outweighs the costs.

The aim of this paper is to present the case study on the implementation of an RBF scheme, to highlight how for pragmatic and operational reasons, implementation may in reality move away from the ideal design and practice, creating a disconnect with RBF’s theory of change which can affect the scheme’s potential for results. In doing this, we focus specifically on a key function of RBF, that of the verification of results described above. As a corollary, our analysis leads to a reflection on how the verification function is operationalized in practice, in terms of implementation challenges, financial and time costs involved and systemic consequences, and whether it responds effectively to what it was originally envisaged. As a case study for our analysis, we look at the RBF scheme implemented as a pilot program in eight districts of Benin, which is further described in the ‘study setting’ section.

The choice of the RBF pilot project in Benin, and of the verification of results carried out within it, does not mean in any way that we consider the scheme, or that component of the scheme, more ridden with problems than others. Indeed, we believe that the situation we describe is quite common in other countries and for the implementation of other schemes (i.e. differently designed RBF schemes, as well as non-RBF projects). We chose this case study because we have been involved, at different levels, in the implementation of the scheme and we believe that the practitioners’ views and experience, although rarely shared beyond reports at national level, are a useful contribution to the academic debate. The fact that that we are able to openly discuss these issues testifies that the project implementation has been an open process by all parties involved (Ministry of Health and donor, in particular), where scrutiny and self-reflection are encouraged in order to learn and improve the scheme.

In the following sections of this article, we first describe the features of the RBF pilot scheme in Benin and in particular the design of the verification processes which are in place at different level. After describing the methods of this research, in the ‘findings’ section, we turn to the verification procedures as implemented in the field, and we examine those practices against the original design as well as against the rationale of the verification and the potential positive effects at system-level identified above.

### Study setting and RBF design in Benin

**Fig. 1:**
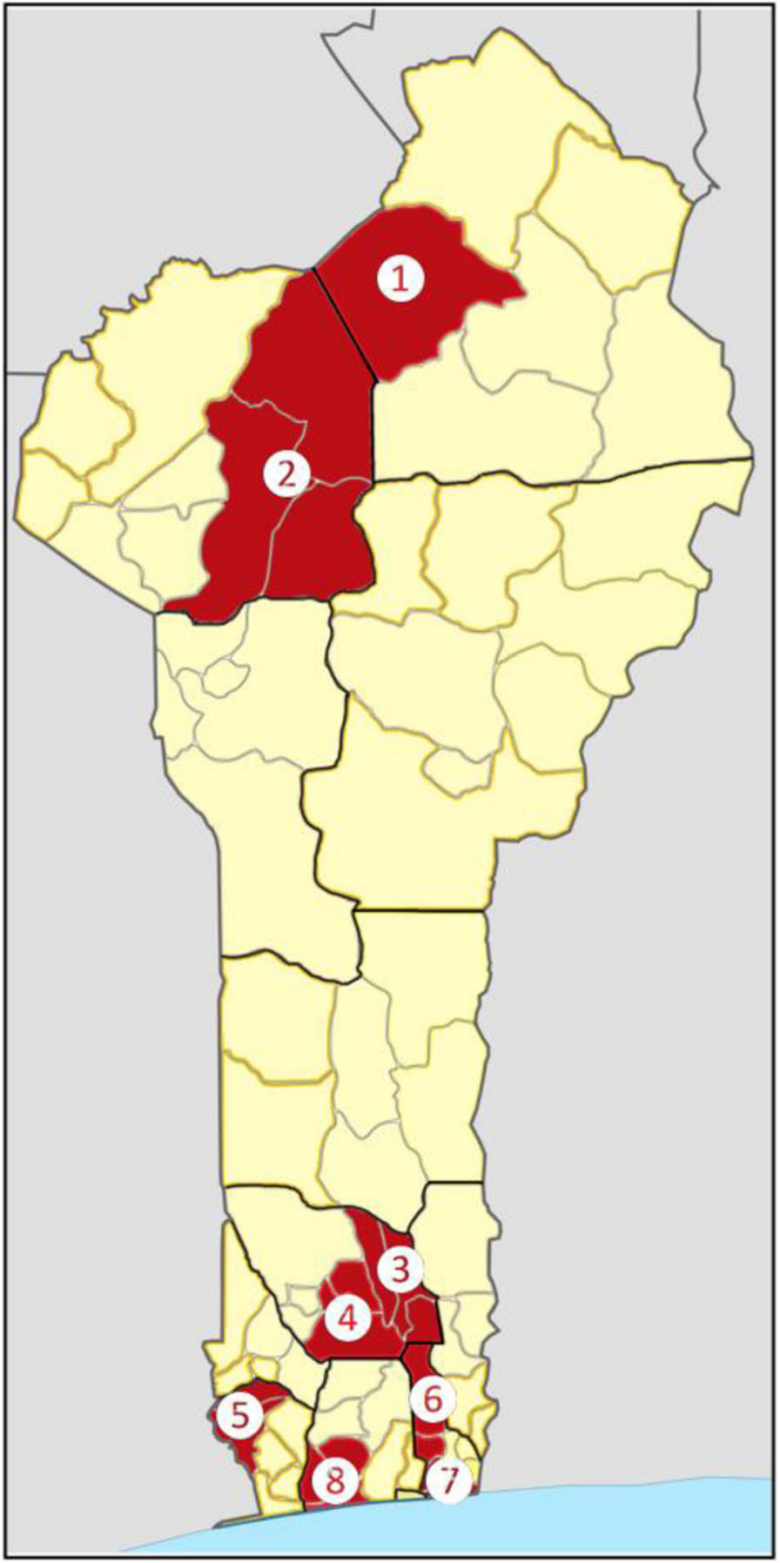
Map of Benin highlighting the 8 districts were RBF was initially introduced

The first RBF pilot effectively started in Benin in March 2012, with funding from the World Bank. Initially, it covered about half of the facilities in 8 districts (*zones de santé*) out of the total 34 of the country, and a population of about 2,377,559 people (23% of the total population) (Fig. 1). The focus of the program is on the health facilities’ productivity and quality of healthcare, and to assess its effectiveness an RCT was planned since its inception and is still ongoing [21]. For the RCT, the remaining facilities in the 8 districts were selected as control and received funds not based on their performance. As of April 2015, RBF has been scaled up to 21 districts, with the support of the Global Fund and GAVI.

#### RBF design and verification of results

To ensure the management of RBF, a Project Coordination Unit was created within the Ministry of Health (MoH), which is in charge of signing the performance contracts with the providers, purchasing the health services (i.e., establishing the list of services included in the scheme and the corresponding bonus), and transferring the payments to the facilities’ bank accounts. Alongside the ministerial Project Coordination Unit, an implementation agency was hired to be specifically in charge of technical assistance, coaching and verification procedures. An international consortium, of which AEDES is part, was selected for this role. The implementation agency consists, in each district, of a team of 2-3 technical assistants, of which one physician with public health experience and 1-2 additional staff according to the number of facilities in the district. At national level, one technical assistance oversees the implementation of the scheme. Technical backstopping is provided by AEDES in Brussels both with regular routine missions and missions to address specific issues.

At the beginning of the project, performance contracts were signed with facilities in both the control and the intervention arm of the RCT (*n* = 188), indicating the quantity and quality indicators included in the scheme, as well as the amount of payment per indicator. Since 2014, quantity indicators include 28 at health center level and 14 at hospital level (Table 1), while the quality checklist is composed of about 100 items, mostly focused on the quality of the health service delivery environment and on the availability of tools and equipment. Verification is performed in both arms, although only the intervention facilities receive a payment based on their performance. Each quarter, a ‘results validation meeting’ is held at central level by the Project Coordination Unit, where the performance of the facilities is reviewed as well as the verification procedures of the implementing agency. Once validated, the performance bonus is transferred to the facilities’ bank accounts by the Project Coordination Unit. Every quarter, a staff meeting is called in facilities to decide the use of funds. Guidelines envisage that facilities must use a minimum of 50% of the bonus to cover recurrent expenditures (such as, drugs, equipment and cleaning materials, small investments, etc.) and the rest (a maximum of 50% of the total bonus) as staff bonus. The individual staff bonus for each health worker is calculated based on their cadre and seniority, as well as on their presence at work for the period considered. Facilities also receive other funds (beyond the RBF bonus) including a fixed budget from the MoH (*crédit delegué de l’Etat*), user fees from patients which are used to fund recurrent costs and purchase drugs [22]. Hospitals are reimbursed for the C-sections which they provide for free under the current governmental policy. In some specific cases, they may also receive reimbursements from health insurance and support (in cash, or kind) from NGOs and associations.

**Table 1:** Indicators included in the RBF pilot in Benin and corresponding payment to facilities

	Health service	Payment 2014 (FCFA)	Payment 2014 (USD)
Health centres (HC)	New case of curative consultation	350	0.71
	New case of curative consultation for poorest in the community (extra payment)	1,750	3.57
	Growth monitoring visit (11-59 months)	420	0.86
	Diagnosis and treatment of malaria in children	330	0.67
	Diagnosis and treatment of malaria in pregnant women	655	1.34
	Diagnosis and treatment of severe malaria in children	5,890	12.02
	Diagnosis and treatment of severe malaria in pregnant women	5,365	10.94
	ANC 1	3,500	7.14
	ANC 1 for poorest women (extra payment)	2,800	5.71
	ANC 4	3,000	6.12
	ANC 4 for poorest women (extra payment)	3,000	6.12
	Normal delivery assisted by skilled personnel	7,500	15.30
	Normal delivery assisted by skilled personnel for poorest women (extra payment)	6,000	12.24
	Postnatal consultation 1 (7th-10th day after delivery)	3,500	7.14
	Postnatal consultation 3 (42nd-45th day after delivery)	1,750	3.57
	New users of long-term family planning (IUD and implant)	6,300	12.85
	New users of short-term family planning	1,750	3.57
	Emergency referral for delivery	3,150	6.43
	Children having received BCG vaccine	875	1.79
	Children having received pentavalent vaccine	700	1.43
	Children fully immunized	3,000	6.12
	Patient referral and arrival to hospital	1,050	2.14
	Detection of TBC+ case	14,350	29.27
	TBC cases treated and healed	15,000	30.60
	Pregnant women detected HIV+ and initiated to ARV treatment	15,750	32.13
	Patients on ARV treatment (first 6 months)	8,750	17.85
	Children eligible to ARV having started treatment	19,250	39.27
	Diagnosis and treatment of STIs	700	1.43
District hospitals	Counter-referral by hospital of patients from HC	3,500	7.14
	Diagnosis and treatment of malaria in children	330	0.67
	Diagnosis and treatment of malaria in pregnant women	655	1.33
	Diagnosis and treatment of severe malaria in children	5,572	11.36
	Diagnosis and treatment of severe malaria in pregnant women	5,075	10.35
	Pregnant women detected HIV+ and initiated to ARV treatment	17,500	35.7
	Patients on ARV treatment (first 6 months)	12,250	24.99
	Children eligible to ARV having started treatment	19,250	39.27
	Dystocic delivery of a patient referred from a HC	17,500	35.7
	Gynecological surgery	26,250	53.55
	Detection of TBC+ case	22,750	46.41
	TBC cases treated and healed	26,250	53.55
	Diagnosis and treatment of STIs	1,750	3.57

Exchange rate (August 2014): 1 F CFA = 0,00204 USD

Similarly to many other RBF schemes in Africa and in particular the early ones in the Great Lakes region [16], the verification of results for the Benin pilot is organized along three main axes [23]. A first verification concerns the quantity of services (among those included in the RBF contract) provided by the facilities and is carried out twice per quarter by the technical assistants of the implementing agency at district level. It aims to confirm the accuracy of the data reported by the facilities in the monthly RBF declarations. In practice, the technical assistants in the district visit each facility every month to compare the numbers detailed in the monthly invoice sent by the facility with those included in the facility registries. It also includes a check of the standards of the service provided. This means that if the service was not provided according to the standards (e.g., the patient receives less than 4 ANC visits and not at the right timing for the ‘ANC 4’ indicator), that particular service will not be counted towards the monthly total and therefore not paid for. Secondly, a verification is conducted to assess the quality of the services provided, using a quality checklist which was prepared drawing from the one used in Burundi [16]. Quality verification is carried out every quarter under the leadership of the District Health Management Team (DHMT - *Equipe d’Encadrement de la Zone Sanitaire* in Benin). The DHMT staff (usually organized two or three teams including 2-3 DHMT staff each) conducts the verification directly for health centers, by visiting each facility in the district and checking the availability of equipment and materials against those indicted in the quality checklist. Although consisting in the same procedure, the quality verification in district hospitals is carried out by peers (i.e. staff from another district hospital). This is because some DHMT staff usually works in the local hospital and therefore would have a conflict of interest in verifying their own service quality. In practice, therefore, each quarter, a team of five from one district hospital is randomly assigned to visit the hospital in another district and fill in the quality checklist. In both cases, the implementation agency supervises the procedures. Finally, a counter-verification or community verification is envisaged to be carried out quarterly by community-based organizations (CBOs). CBOs are selected by the central Project Coordination Unit among NGOs with a strong presence in at least one of the pilot districts, which are independent, have no connections with health facilities, have experience in carrying out surveys and can hire enumerators, have a bank account, and exist since at least two years. In those districts, they contract enumerations to visit communities with the aim of tracing some of the patients who visited the facility within the communities, and check (i) their actual existence and that they received the service as indicated, and (ii) their level of satisfaction with the healthcare services. A random sample of the patients is prepared by the implementation agency. Additionally, CBO’s enumerators are also supposed to carry out unannounced visits to the facilities each semester to assess the waiting time and the quality of patient reception.

## Methods

This study takes the approach and methods of practical ‘action research’. Action research is essentially “concerned with generating knowledge about a social system, while, at the same time, attempting to change it” [24]. It usually aims to generate understanding and improvements in practice, and is undertaken by and with those who will take action to ensure such changes [25]. In our case, the reason for choosing this approach lies is the fact that the research was initiated as a reflective process conducted in parallel to operational work with the aim of identifying problems and challenges, and providing practical solutions to them, carried out by individuals working within the implementation team. Indeed, all authors have worked at some point in time to provide technical support to the RBF pilot scheme, although at different degrees ranging from a brief involvement at the beginning of the project (two weeks in 2012 for MPB and OB), to a continuous support with several missions from July 2014 to the present date for MA. Because of our role with the implementation agency, we had access to data, documents and information which were collected for the daily management of the project, as well as for the ‘documentation’ process which took place alongside the implementation. This study makes use of the quantitative and qualitative information collected, both at central level in Cotonou and in the districts where the RBF project is implemented, for the ‘documentation’ of the project, but, importantly, all information has been fully re-analyzed for the purpose of the present study.

Our participant observation during the project’s implementation and contextual knowledge helped shape the research questions and the research design, as well as provide information for our analysis and data interpretation. In addition, to inform our analysis, we reviewed the existing documents and published literature, both referring to the RBF pilot project in Benin (e.g., project documents and technical reports), as well as to RBF theory and practice in other countries, with a particular focus on the verification function. Documents reviewed include the published literature, as well as the grey literature available from RBF websites, such as the online group of the Performance-Based Financing Community of Practice in Africa^1^ and the RBF website of the World Bank^2^. Finally, as detailed above, we make extensive use of secondary data, collected for the day-to-day activities and documentation of the project, which we re-analyzed in anonymized form specifically for this paper. Secondary data include quantitative information, such as (i) RBF data on service outputs (which are publicly available from Open RBF^3^) and data on the outcome of the verification procedures from 2012 to 2015, (ii) information on budgets and financial costs, also from 2012 to 2015, (iii) information on time use (in particular on verification procedures) by the technical assistants working for the implementation agency in the pilot districts. The latter was collected through daily timesheets completed by all the technical assistants (*n* = 20) from June to August 2015. Secondary qualitative data include the information contained in a series of focus groups discussions (FGDs) with CBOs (*n* = 5) and informal interviews with district medical officers (*n* = 5) and technical assistants (*n* = 8), previously carried out for the operational work. Given the study approach, both FDGs and interviews took an informal approach and were not recorded and transcribed verbatim, but rather summarized in the form of field notes [26], which were later analyzed, with reference specifically to the issue of verification. Similarly, our participant observation was unstructured and observations fed into field notes used initially for documentation purposes and re-analyzed for this study. Qualitative data were manually analyzed using a series of pre-identified themes. These themes focused on the potential challenges linked to the different elements of the verification, and included: (i) workload for implementing agency and DHMTs; (ii) analysis of verification data; (iii) cost of verification; (iv) community verification procedures; (v) selection and management of CBOs. For each of these, we also focused on the consequences that they have on key components of the project, which may affect the underlying theory of change of RBF. Secondary analysis was performed on quantitative data already available to triangulate and further explore issues emerging from the qualitative analysis. In particular, data were used to calculate (i) outcomes of the verification, (ii) technical assistants’ time use, (iii) delays between service delivery, verification and payment, (iv) costs of verification.

We are aware of the potential limitations of the methodology of our research, which stem from the closeness of our perspective with the subject of study. In order to mitigate the possible issues, we took them constantly in consideration when designing and carrying out the research, by actively exercising reflexivity and openly reflecting on our positionality as participant observers to different extents [27]. Dialogue among the authors, whose degree of involvement with the projects vary so that both insider and outsider views are represented, as well as with others within and outside the project has enriched our data analysis, findings and interpretations. On the other hand, our position also bears the advantage of allowing detailed knowledge of the context and the implementation processes, including on challenges that may be difficult to see by external observers, as well as it provided us with access to data that may be otherwise complex to obtain. Finally, we have been open among ourselves and with those who provided us with comments and reviews about the potential conflict of interest, stemming from the fact that we are involved in the implementation of the project (and therefore likely to be interested in providing a positive imagine of it), as well as producing research on it. We believe that the constructive critic that we move to the verification process in our findings and discussion sections shows our impartiality towards the project’s assessment and confirms our objective (alongside that of our funders and of the Ministry of Health) of raising important operational issues regarding RBF which are often overlooked in the theoretical literature and produce a rigorous account of them, with the final aim of improving RBF schemes in Benin and elsewhere.

### Results

#### Outcomes of the verification procedures

First, we briefly present the outcome of the verification process in terms of what were the results of the various verification procedures at facility level. In terms of quantitative verification, for the duration of the project (2012–2015), based on the analysis of Open RBF data, we found that the discrepancy between service volume declared by the facilities and verified data greatly varied according to the indicator (from 4% as an average for ‘new users of family planning’ to 51% for ‘patient referred to hospital’). For any specific indicator, the discrepancy remain generally stable overtime, with some indicators (such as, patients referred to hospital) more prone to errors or frauds than others. We also found that some facilities and districts were more prone to higher discrepancies than others. Regarding the quality verification, scores greatly varied between health facilities. Thus, in the third quarter of 2015, these scores ranged from 4% to 96% according to the health centers, with an average at 68.7% and a median of 71.8%. During the RBF project (between the first quarter of 2012 and the last quarter of 2015), quality scores increased from 39.3% to 71.4% for health centers and from 52.4% to 84.9% for district hospitals. Last, we found that data produced by the community verification were less often collected than originally envisaged and, as important, that those data were not systematically analyzed. Partial analysis of the number of patients missing from community tracing shows them between 12% and 47% during the third quarter of 2013 (first community survey). However, other data on patients’ satisfaction were not analyzed at all so that aggregated figures are not available, while unannounced visits to facilities were never carried out.

#### Implementation of the verification procedures

In any RBF project, data and analysis resulting from the verification procedures allow not only detecting errors or frauds, but also calculating payments. In a study perspective, the verification outcomes described above also provide a first understanding of the verification procedures and of their challenges. In this section, we look at the implementation processes in order to explore how the actual practice can differ from what was initially envisaged.

**Fig. 2:**
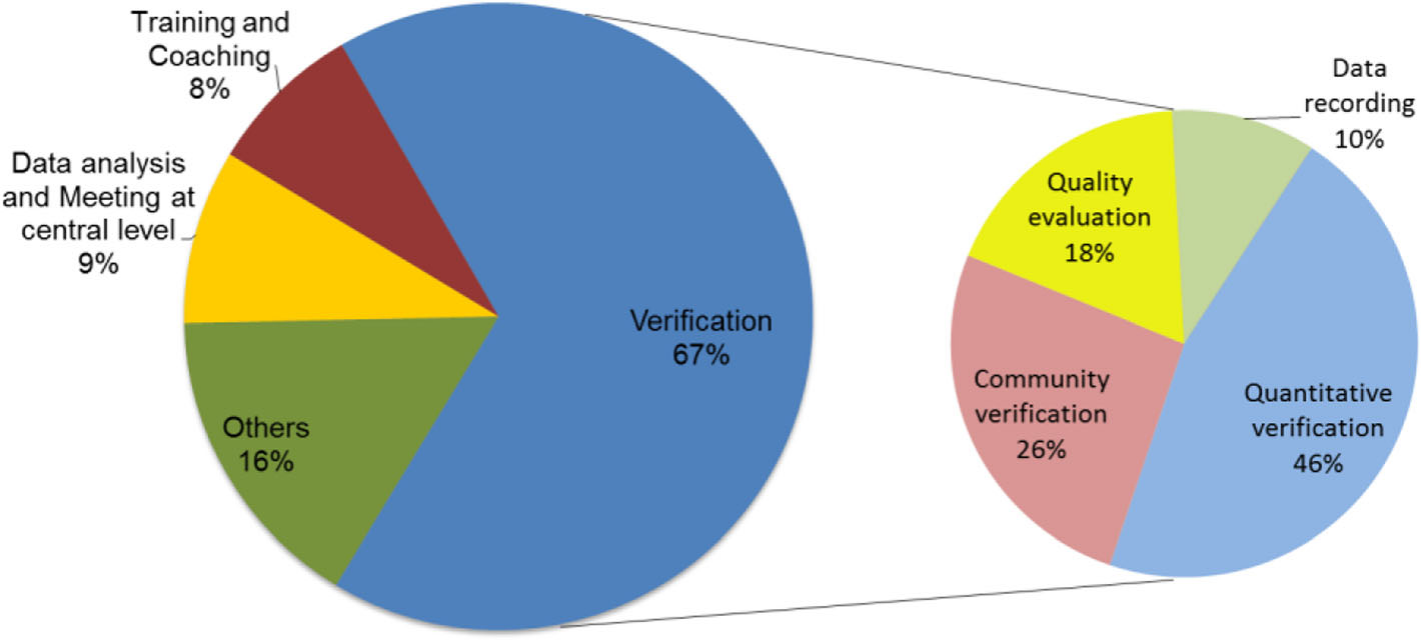
Proportion of time spent on different activities for implementation agency’s staff in the field

From the reviews of documents describing the design of the verification procedures (e.g., implementation manuals), it appears that the processes in place to verify the services provided is complex and lengthy and relies heavily on the implementation agency’s technical assistants. Indeed, it emerged that, in the project design, the implementation agency, through the technical assistants at district level, plays a particularly important role in all the three axes of the verification process. First, the agency is fully in charge of the quantity verification; second, through the supervision of the entire process it also contributes to the quality verification under the DHMT leadership; third, it organizes and supervises patient tracing by CBOs. Additionally, implementation agency’s staff is also in charge of data entering into the Open RBF information system. And last, the agency is responsible for payment calculations, which are then approved by the Project Coordinating Unit in charge of making the payment to health facilities. The long, complex and time-consuming features of the verification processes were also confirmed by the interviews with the technical assistants of the implementation agency, during which the issue emerged repeatedly and was a clear source of frustration. In order to triangulate the qualitative information on this point, we analyzed the timesheets filled in by the implementation agency staff in the districts between June and August 2015. We found that verification procedures take up 67% of their time, of which 46% for quantitative verification, 26% to support the qualitative evaluation and 18% to prepare and supervise the community verification carried out by CBOs. The remaining time (10%) is spent on data entering and recording (Fig. 2). Data from timesheets also show that verification activities become particularly time-consuming after the end of each trimester when all verification procedures and payment calculations need to be carried out.

During the informal interviews, technical assistants at district level not only vented their frustration concerning the time spent of the verification procedures, but also described the consequences of it. They noted that the time spent on verification yielded to a loss in the quality of their work and that furthermore that it is done at the expense of activities that they consider being more important and constructive and adapted to their skills, such as data analysis, one-to-one coaching and comprehensive feedback to providers. As a consequence, the lack of time available for feedback to health facilities staff seems to have important draw backs on the potential of RBF to actually impact on facilities’ performance (previously presented as one of the possible positive spill-over effects of the verification and data availability).

Competition of time is not only an issue for the implementation agency’s staff, but was also raised as an issue for DHMTs during interviews with the district medical officers (DMOs). Under RBF, DHMTs are supposed to lead the qualitative verification process as well as support in the elaboration of facilities’ business plans and coaching of health facilities’ staff. However, as DMOs stressed, aside the RBF-related duties, DHMTs have a large number of other routine tasks to perform, such as supervision, monitoring and routine reporting, for which they are directly accountable to their hierarchy. As activities related to RBF are poorly integrated with those activities, DHMTs happen to give a lower priority to the RBF verification tasks. Moreover, RBF is often in direct competition with activities such as those related to vertical programs (e.g. meetings, training or national prevention campaigns), that generally entail the payment of per diems. While it is true that DHMTs also receive daily allowances for RBF qualitative verification, our data on payments show that the latter are generally of a lower amount and less readily paid. This fact is likely to contribute to DHMTs’ low motivation to be involved in RBF-related activities. Additionally, DMOs also mentioned that sometimes their offices lack equipment (e.g. vehicles) or commodities (such as fuel) to carry out verification activities. In either case, because of the DHMTs’ insufficient time or lack of incentives and resources, this has resulted, in the Benin’s project, in delays in carrying out the quality verification at facility level, with consequent postponements of RBF bonus payments to facilities (as shown by the analysis of payment delays below). Additionally, the limited involvement of the DHMTs and their incapacity to take real leadership in the verification process hampers the potential positive effects of RBF on the governance and stewardship at district level.

For what concerns the community verification procedures, during the FGDs, the CBOs’ enumerators in charge of it pointed to the challenges that they face in tracing patients in communities, especially in the rural areas of the northern part of the country, which requires extensive travel. An additional challenge was pointed out by the technical assistants during the interviews and concerns the way the verification process is organized and how incentives are set. Indeed, the technical assistants observed that, while the CBOs that run the community verification are paid a fixed lump sum regardless of the amount of work they effectively carry out (i.e. number of patients traced), the hired enumerators are insufficiently paid to cover their running costs and therefore are not sufficiently motivated to carry out their tasks effectively. Another frustration and challenge for the technical assistants relates to the fact that, due to the design of the contracts, CBOs are not accountable to the implementation agency even if they are supposed to be supported and supervised by it. As a consequence, the implementation agency has greater difficulties to enforce standards for reporting and scheduling. Another important point raised is that CBOs recruited for this task are in fact national NGOs, organized in a hierarchical way, with headquarters in Cotonou. This organizational feature raises questions about their actual relationship with the communities whose voice they are supposed to convey. Therefore, it seems that the system could be potentially taken advantage by elites in the capital rather than by preexisting local organizations, reflecting the idea of a community-owned process and representation.

Finally, during fieldwork, we noted that, once community-based data are collected, there is little or no analysis performed, and no sanctions are applied to providers based on the detection of frauds. Besides, in the Benin’s RBF scheme, it was not planned to use patient satisfaction for rewards or sanctions. This issue does not only concern the community verification, but all the components of verification, because of the reticence to apply sanctions to the cases of over-reporting of number of services provided. Through the review of reports and minutes of the results validation meetings held in Cotonou, we found that in many instances, an approach based on dialogue was preferred to the direct application of pecuniary sanctions. Importantly, the preference for dialogue over sanctions not only weakens the power of incentives, but also renders the verification process (and the money and time spent on it) practically irrelevant.

By analyzing existing activity reports and payment documents, we found that a critical consequence of the complexity of the verification procedures as described above, and of the challenges for its operationalization, is the important delay of the payment cycle. The lag time between the verification and invoice transmission were also compounded by the lengthy procedures at national level for the calculation and execution of the payments to the health facilities’ bank accounts. Table 2 below presents the delays between the invoice transmission at the end of the verification procedures and the actual bank transfers. It shows that delays can be as long as eight months from service provision to payment. Once again, in terms of the RBF theory of change, this issue engenders a disconnect between the effort of the providers (reflected in the quantity and quality of services provided) and the reward. As the link between payment and performance becomes less evident, the potential of the performance-contract to incentivize workers becomes weaker.

**Table 2:** Delay between service provision, verified invoice transmission and RBF bonus payment

Quarter	Quarter end	Verified Invoice transmission	*Delay (months)*	Bank transfer	Delay (months)	*Total delay (months)*
Q2 2012	Jun-12	Sep-12	3.5	Nov-12	1.5	5
Q3 2012	Sep-12	Dec-12	3.5	Apr-13	3	6.5
Q4 2012	Dec-12	Apr-13	4.0	Jul-13	3.5	7.5
Q1 2013	Mar-13	Jul-13	4.5	Nov-13	3.5	8
Q2 2013	Jun-13	Sep-13	3.5	Dec-13	3	6.5
Q3 2013	Sep-13	Dec-13	3.5	Feb-14	2	5.5
Q4 2013	Dec-13	Apr-14	4.0	May-14	0.5	4.5
Q1 2014	Mar-14	Jun-14	3.5	Jul-14	0	3.5
Q2 2014	Jun-14	Sep-14	3.5	Jan-15	3.5	7.0
Q3 2014	Sep-14	Dec-14	3.0	Feb-15	2	5

#### Costs of verification processes

By using the project’s budget data, our analysis was also able to assess the costs of the verification processes. The cost calculations presented below include only the financial costs (e.g., financial transactions that are a results of the verification activities introduced by RBF) and do not include economic costs (e.g., also the time spent by DHMTs and implementing agency staff on verification), nor the capital costs [20]. It emerges that, while about 1,936,075 USD were provided to facilities as RBF bonus in the period between July 2013 and June 2014, in the same period about 958,484 USD were spent for the verification procedures. This means that for each 1 USD paid to the providers, about 0.50 USD were used for the verification, of which 39% goes to the implementing agency and 61% to the CBOs in charge of the community verification.

## Discussion

This paper aims at describing the implementation practices of an RBF scheme in order to show that the actual practices can greatly differ from the ideal design and therefore result in a disconnect with the theory of change underlying RBF itself. Logically this may have important consequences on RBF’s potential to generate the envisaged change and on the local health system. To demonstrate that, we use the case of the verification of results in place in the RBF pilot in Benin. Our findings specifically highlight three main points, concerning the verification of results, but also more broadly at operational and methodological levels.

First, verification is known to be a critical component of RBF and a key element in its theory of change, as it should (i) ensure a transparent and timely payment to providers, (ii) avoid fraud and provide a real threat of sanction, (iii) generate data which can be analyzed and fed back to providers and managers, (iv) improve governance and stewardship at district level, (v) channel the voice of patients and communities. After the scrutiny of the implementation practices in Benin, however, it emerges that very few of these features are in place and verification processes are ridden with challenges. In particular, the verification articulated along the three axes as described seems complex and time-consuming, which has consequences in terms of workload for actors at local level (both of the implementing agency and DHMTs), lack of data analysis and information feedback, and delayed payments, and therefore in practice too often ends up differing from what envisaged at design stage. Importantly, we also find that the costs of the verification, and in particular the cost of the community verification, is high as compared to the funds disbursed to the service providers.

These findings on verification costs, but also on lack of adherence to design, payment delays and concerning the specific challenges of community verification are not dissimilar to those of studies carried out in other settings. In Burundi, for example, a study [19] found that verification activities make up 16% of the total costs of the national RBF project and that verification costed 25-30% of the entire budget at pilot stage, when the scheme was NGO-managed. The same study in Burundi also stressed that the role of verification outcomes as sanction for providers has a marginal impact on the scheme, as sanctions are rare and partially applied, and, similarly to Benin, data are still not fully exploited for analysis and feedback [19]. The problem of delays between service delivery and payment of providers is also common across RBF projects in different settings, because of difficulties in the verification processes and/or in the disbursement procedures. Such delays, that we found for the case of Benin, have also been documented in Sierra Leone [28], Nigeria [29, 30] and Uganda [12], and have a critical impact on the potential for results as they delink performance and payment. Regarding the community verification, many of the problems pointed to by our analysis in terms of costs and practical feasibility were also made in a debate among members of the Community of Practice with reference to Burkina Faso and other countries (DR Congo, Rwanda, Central African Republic, and Haiti) [31]. In terms of the relevance of the community verification, previous research has highlighted the dangers of conflating community verification with a form of community participation, as the outcome of that verification only represents the views of few users and CBOs lack the ability to enforce real changes at facility level [32]. In Benin, this issue is further compounded by the elite appropriation, as CBOs are in fact powerful national NGOs which resort to hiring staff (not necessarily from the communities) to conduct patients’ tracing surveys.

Unsurprisingly, the challenges in the verification process affects RBF’s potential for results and hamper the hoped-for positive effects on the wider health system, as envisaged by the underlying theory of change. However, despite the challenges, verification is an essential element of RBF, especially at the beginning of the program when actors were not yet familiar with the new institutional arrangements in place as it ensures the credibility of RBF with all stakeholders. As a consequence, verification procedures cannot be simply scraped away, but must be actively adapted to context to make sure that they are in line with RBF’s theory of change, as well as operationally feasible and financially viable. In particular, there is the need to find a balance between rigorous verification processes and their practical feasibility and costs in terms of financial resources and time, which would be otherwise available for other activities including to provide funds to providers. The challenges we encountered in the implementation question whether the rationale for thorough and expensive verification is still valid, or rather other solutions and adaptations can be proposed. Indeed, some of the issues detailed in this article have been now successfully addressed in Benin through the constructive collaboration between Project Coordination Unit of the MoH, the main donor and the implementing agency. For instance, given the cost of CBOs and lack of use of the data, community verification was suspended while a more adequate procedure is being design. With reference to other countries and organizations, a collection of case studies, prepared by the World Bank, describes the verification procedures in a number of RBF schemes globally and provides useful information on their results and the implementation challenges [33, 34]. From that analysis, as well as from our study, a strong argument in favor of ‘risk-based verification’ emerges. This refers to a verification which is not systematic and comprehensive, but focused on certain indicators (higher volumes, complex indicators, higher payments) and certain providers (random selection, higher volumes, performance outliers) [34]. Additionally, future positive developments in the verification may include the use of new technologies such as use of IT devices to﻿ trace﻿ patients, such as mobile phone surveys, as well as tablets for easier, more rapid and cost-effective data recording and analysis.

Secondly, and broadening our first point, our analysis shows that RBF should be carefully tailored to the context in each of its operational components (including verification, but also beyond it), and space should be allow for iterative adjustments during the implementation, especially at pilot stage but also when scaled-up. Such iterative adjustments will ensure that the RBF project’s operationalization is feasible and optimal, given the local features and the available financial resources, time and skills. This should avoid that the practical challenges create a complete disconnect between what is implemented and the key elements underlying the rationale and the theory of change of the RBF intervention. While in the early years of RBF implementation, the focus of most experts and practitioners has been on how RBF *should* be designed, as such establishing a certain ‘orthodoxy’ for example through the PBF Community of Practice, in the last few years, the literature on the influence of context and implementation on RBF results has been growing and there is an increasing recognition among researches and practitioners that the local challenges in the operationalization of RBF must be taken actively into account, both in the design of RBF, which should reflect and be adapted to the specificities of the context, and in the implementation, which should remain flexible and adapted iteratively, especially at pilot stage [35].

Finally, from a methodological perspective, our study confirms the importance of including in the analysis of RBF interventions not only their end-point results (whether service outputs or health outcomes), but also the processes through which these results are achieved, and in particular carefully scrutinizing implementation practices to complement impact evaluations [12, 15, 28, 30]. It seems that RCTs often assume clear-cut design and perfect fidelity in implementation which would allow to clearly link results to specific components of the intervention and test the correctness of its underlying assumptions. However, in practice, at implementation stage, logistics and practical questions, scarcity of time and funds as well as local political economies and cultural features often substantially modify the RBF’s design. As a consequence, the results of the impact evaluations are difficult to interpret as a neat evaluation of the theoretical mechanisms underlying the RBF intervention, but rather must be explained with reference to the implementation processes and the broader context [36].

## Conclusions

This study illustrates, through the example of the verification of results in an RBF pilot project in Benin, that as practitioners, researchers, funders and policy-makers, we should collectively pay more attention to the operational components of RBF schemes. In RBF (as in any complex health system interventions), “the devil is in the detail” as some RBF experts like to say. Our study suggests that there is a need to focus on the details of the design of RBF interventions to avoid standardization and ‘copy-paste’ approaches and better adapt it to the local context, as well as on the details of the implementation to make sure that it is feasible and effective, and ensures an alignment with the underlying theory of change of RBF. Moreover, the scheme should be regularly and iteratively revised to guarantee the relevance of all its components during the actual implementation phase.

Methodologically, we need to go beyond the exclusive focus on the impact of RBF to look also at other elements. As stress by others, context and implementation are key [30], and it is also critical to examine the consequences of the RBF schemes as actually implemented on the health system more broadly [11]. In this sense, we believe that the views of the implementers and practitioners in the field, although often limited to internal documents and discussions and rarely reported in the published literature, can provide useful insights and an operational perspective which complements the more traditional impact evaluations and academic studies.

## Abbreviations

AEDES: Agence européenne de développement et santé
ANC: Antenatal care
ARV: Antiretroviral treatment
CBO: Community based organization
DHMT: District health medical team
DMO: District medical officer
F CFA: West Africa Franc (Benin currency)
FGD: Focus group discussion
GAVI: Global alliance for vaccine and immunization
HC: Health centre
MoH: Ministry of health
NGO: Non-governmental organization
RBF: Results-based financing
RCT: Randomized control trial
STI: Sexually transmitted infection
TBC: Tuberculosis
USD: United States Dollar

## Footnotes

1
https://groups.google.com/forum/#!forum/performance-based-financing

2
https://www.rbfhealth.org/

3
http://www.beninfbr.org/
